# Differential plasma microvesicle and brain profiles of microRNA in experimental cerebral malaria

**DOI:** 10.1186/s12936-018-2330-5

**Published:** 2018-05-11

**Authors:** Amy Cohen, Anna Zinger, Natalia Tiberti, Georges E. R. Grau, Valery Combes

**Affiliations:** 1 Vascular Immunology Unit, Department of Pathology, The University of Sydney, Sydney, Australia; 2 School of Life Sciences, Faculty of Sciences, University of Technology, Sydney, Australia; 3 La Jolla Infectious Diseases Institute, San Diego, CA USA

**Keywords:** Malaria, Cerebral malaria, microRNA, Disease severity, Biomarker, Microarray, Pathogenesis

## Abstract

**Background:**

Cerebral malaria (CM) is a fatal complication of *Plasmodium* infection, mostly affecting children under the age of five in the sub-Saharan African region. CM pathogenesis remains incompletely understood, although sequestered infected red blood cells, inflammatory cells aggregating in the cerebral blood vessels, and the microvesicles (MV) that they release in the circulation, have been implicated. Plasma MV numbers increase in CM patients and in the murine model, where blocking their release, genetically or pharmacologically, protects against brain pathology, suggesting a role of MV in CM neuropathogenesis. In this work, the microRNA (miRNA) cargo of MV is defined for the first time during experimental CM with the overarching hypothesis that this characterization could help understand CM pathogenesis.

**Results:**

The change in abundance of miRNA was studied following infection of CBA mice with *Plasmodium berghei* ANKA strain (causing experimental CM), and *Plasmodium yoelii*, which causes severe malaria without cerebral complications, termed non-CM (NCM). miRNA expression was analyzed using microarrays to compare MV from healthy (NI) and CM mice, yielding several miRNA of interest. The differential expression profiles of these selected miRNA (miR-146a, miR-150, miR-193b, miR-205, miR-215, miR-467a, and miR-486) were analyzed in mouse MV, MV-free plasma, and brain tissue by quantitative reverse transcription PCR (RT-qPCR). Two miRNA—miR-146a and miR-193b—were confirmed as differentially abundant in MV from CM mice, compared with NCM and NI mice. These miRNA have been shown to play various roles in inflammation, and their dysregulation during CM may be critical for triggering the neurological syndrome via regulation of their potential downstream targets.

**Conclusions:**

These data suggest that, in the mouse model at least, miRNA may have a regulatory role in the pathogenesis of severe malaria.

**Electronic supplementary material:**

The online version of this article (10.1186/s12936-018-2330-5) contains supplementary material, which is available to authorized users.

## Background

Malaria is a devastating infectious disease transmitted to vertebrate hosts by the bite of *Plasmodium*-infected female *Anopheles* mosquitoes [1]. Malaria remains a substantial problem affecting humans today, with approximately half of the world’s population at risk. Every year, around 200 million people are infected by *Plasmodium falciparum* alone worldwide [2]. Severe malaria is most commonly caused by infection with *P. falciparum*, and cerebral malaria (CM) is the most severe manifestation of malaria infection. CM is a syndrome characterized by unarousable coma and unconsciousness, often leading to death, or the occurrence of neurological sequelae in survivors [3]. Approximately 1% of individuals with malaria infection develop CM; however, the reason for this is currently unknown. The pathology of this syndrome is still incompletely understood, as the existing hypotheses cannot fully explain all manifestations and clinical signs of CM [4]. Other manifestations of severe malaria can result in severe anaemia and respiratory distress, termed here non-cerebral severe malaria (NCM) [2]. Current treatment options for CM—that is, anti-malarial medications combined with immediate intensive care—have an 80–85% success rate. However, this is not the case in many parts of Africa, where there is increasing levels of drug resistance and, in some cases, insufficient access to adequate hospital care [5]. In fact, despite successful prevention, persisting neurological sequelae and long-term impairments are present in 25% of paediatric CM cases [6]. Therefore, further research into the pathology, progression, and treatment is essential in the prevention and elimination of malaria.

Of recent interest is the role that microvesicles (MV) play in the pathogenesis of CM both as markers of severity and effectors. Plasma MV are submicron vesicles (diameters of 200–1000 nm) released from most cell types in the microvasculature, including those involved in the pathogenesis of CM, such as platelets, erythrocytes, endothelial cells (EC), and leucocytes [7]. They form through the budding of the plasma membrane and take with them cytoplasmic proteins, lipids, and nucleic acids, as well as surface antigens from the cell of origin. Consequently, they are present in the circulation and can bind to target cells, propagating biological signals and participating in cell–cell communication [8]. While circulating MV play a role in maintaining homoeostasis at normal physiological levels [9], imbalances in their numbers have been shown to be associated with pathophysiological conditions [7]. During CM, higher levels of MV correlate with the presence of neurological symptoms, increased disease severity, and coma depth in human patients [10, 11] and the murine model [7, 12, 13], while in NCM infections, the same significant increase is not observed [12, 13]. In addition, blocking the production of MV, genetically [12] or pharmacologically [14], is associated with protection against the development of experimental CM. In mice, MV accumulate within the brain microvessels of infected but not healthy recipient mice, following adoptive transfer [13]. Analysis of MV, protein, lipid, and nucleic acid content provides a strong basis for studies to understand their biology and pathophysiology [15, 16]. In disease states, MV derived from the injured organ likely contain valuable biomarkers, including miRNA, for determining the site, type, and extent of disease pathology [17].

miRNA are small, single-stranded non-coding RNA that control more than 30% of protein-coding genes, through post-transcriptional regulation of targeted gene expression and RNA silencing [18]. miRNA are known to play key regulatory roles in numerous biological processes, including cell proliferation, development, differentiation, and apoptosis [19]. miRNA were first characterized within circulating plasma vesicles by Hunter et al. [20]. Further to this, miRNA contained within MV were shown to be transferred to target cells through the circulation, suggesting that the biological effect of cells may, at least partially, depend on MV-shuttled miRNA [21].

miRNA have been shown to be dysregulated in a range of parasitic diseases [22]. Specifically, in malaria infections within mammalian hosts, several studies have explored miRNA changes before and after *Plasmodium* infection in different tissues, including liver and brain [23–26], and within circulating extracellular vesicles—both MV and exosomes [27–29]. Studies using the murine model have investigated the effect of *Plasmodium* infection on miRNA signatures within these tissues and plasma, and found that miR-16 and miR-451 decrease in abundance, while miR-27a and miR-150 increase in response to infection, and that blocking some of these miRNA confers protection to those mice. In contrast, miR-451 is increased in extracellular vesicles from infected red blood cells [27]. These changes in miRNA abundance are reversed following anti-malarial treatment [30–32]. These studies suggest that miRNA could be involved in the protective immune response or resistance against malarial infection, as well as in CM pathogenesis, depending on their downstream targets and abundance during infection. In addition, there is evidence of the impact that interactions between parasite, host, and vector can have on miRNA abundance during malarial transmission [33–36].

Despite these studies, an unbiased high-throughput characterization of miRNA abundance in MV during CM has not yet been carried out. This study examines the abundance of miRNA carried within plasma MV during experimental CM and NCM, as well as in non-infected (NI) mice. MV miRNA are then compared to those circulating in MV-free plasma and expressed in brain tissue, showing that, following infection, specific changes are observed in the three types of samples. These changes provide potential novel avenues of research into further understanding the pathogenesis of severe malaria with a particular focus on CM, in order to harness this information for therapeutic purposes.

## Methods

### Mice

7–10-week-old CBA mice were obtained from the Animal Resource Centre (Perth) and housed under pathogen-free conditions, as per approved protocols of the University of Sydney Animal Ethics Committee (protocol number 2015/832 and 2013/5317). For experimental comparisons, three biological groups of mice were used: non-infected (NI), CM caused by infection with the *Plasmodium berghei* ANKA strain, and NCM caused by *Plasmodium yoelii* infection. As previously described, CBA mice are susceptible to *P. berghei* infection and succumb to CM during the neurological phase, between day 6 and day 14 post-infection (p.i.) [37]. Mice infected with *P. yoelii* survived beyond this point and display severe anaemia in the 3rd week after infection.

### *Plasmodium* inoculation

All infections were initiated by intraperitoneal injection of 1 × 10^6^ *P. berghei* or *P. yoelii*-parasitized red blood cells (pRBCs), as previously described [38]. Parasitaemia was monitored by counting pRBCs in Diff-Quick-stained (ProSciTech) thin blood smears by light microscopy on day 4 p.i. and every 1–2 days after this, for the duration of the infection. Mice were assessed using a clinical evaluation score, and CM diagnosed if mice presented with ruffled fur, severe motor impairment or convulsions, and were given a score of 3 or 4 as previously described [39]. *P. berghei*-infected mice were sacrificed at the time of CM (typically day 7–8 p.i.), and *P. yoelii*-infected mice without any of the signs mentioned above were classified as NCM, and sacrificed at the same time, with comparable levels of parasitaemia of approximately 7% [24].

### Blood sampling, MV purification, and brain tissue preparation

Mice from all biological groups were sacrificed, and blood and brain tissue were collected. Mouse venous blood was collected by retro-orbital puncture under anaesthesia in 0.129 mol/L sodium citrate (ratio of blood to anticoagulant 4:1). Platelet-free plasma (PFP) was prepared as previously described [13]. PFP was then centrifuged at 18000*g* for 45 min and the supernatant removed and retained (MV-free plasma), while the pellet was centrifuged for a further 45 min at 18000*g* in a solution of PBS and sodium citrate (ratio of PBS to anticoagulant 3:1) to pellet MV. Whole brain tissue from each mouse was placed immediately in RNAzol (Molecular Research Centre, Inc.) and homogenized. MV, MV-free plasma, and homogenized brain tissue samples were stored at − 80 °C until RNA extraction was performed. 100 mg of homogenized brain tissue was used for RNA extractions, as per the manufacturer’s instructions. For microarray analysis, MV samples were pooled during vesicle purification; all samples for RT-qPCR analysis were taken from individual mice and kept separate.

### RNA extraction

Total RNA was extracted from pelleted MV, 0.4 mL MV-free plasma, and 100 mg brain tissue using the commercially available protocol from Molecular Research Centre Inc. A phase separation step using 4-bromoanisole (MRC) was added to optimize RNA preparation from low amounts of starting material. All centrifugation steps were performed at 4 °C, and precipitation was performed overnight at − 20 °C, with glycogen added to preserve small RNA. The concentration of RNA was determined using Nanodrop ND-1000 spectrophotometry (Nanodrop Tech), and the purity was assessed by calculation of the ratio of absorbance at 260 and 280 nm, with a cut-off of 1.6 used. RNA concentrations of approximately 30–150, 50–300, and 3000–9000 ng/μL were achieved from MV, MV-free plasma, and brain tissue, respectively.

### cDNA synthesis and preamplification

For OpenArray analysis the starting RNA concentration was set at 100 ng; for RT-qPCR analysis, it was 10 ng. For both, preamplification was used to increase the amount of cDNA. cDNA synthesis and preamplification were performed using commercial rodent kits (Megaplex RT and preamplification kits) and rodent Megaplex™ Primer Pools for OpenArray, or custom primer pools of the targets of interest described below, for RT-qPCR (ThermoFisher Scientific).

### OpenArray analysis

Pre-amplified samples were loaded onto TaqMan OpenArray MicroRNA Panels to be run on the QuantStudio 12K Flex system, as per the manufacturer’s instructions (ThermoFisher Scientific). 754 rodent miRNA, consisting of validated mature mouse and rat miRNA (Sanger miRBase release v.15), were amplified in each sample, with technical replicates, together with internal controls. QuantStudio and ExpressionSuite Analysis software (ThermoFisher Scientific), utilizing the comparative (ΔΔ) C_T_ method, were used to quantify relative expression across all miRNA and samples with relative quantification values expressed as CM/NI. Global normalization was performed using the global mean CT value of targets common to every sample as the normalization factor, on a per sample basis—the recommended and most robust strategy for normalization in large scale (> 384) miRNA expression profiling studies [40]. To reduce false positive results, C_RT_ (cycle of relative threshold) values were filtered to remove values over 30, considered not to be true results. In addition, technical replicates with a standard deviation (SD) over 0.5 between C_RT_ values, and with an amplification score below 1.2 (low quality) were removed. Lastly, miRNA amplified in less than 50% of biological replicates were considered as lowly expressed and excluded from the analysis. Relative quantification was calculated using algorithms included in the ExpressionSuite analysis software. Statistical calculations were performed using multiple t tests—one per row, with fewer assumptions.

### Pathway analysis

Microarray results were analyzed with DIANA miRPath version 3 [41] and Ingenuity^®^ Pathway analysis—IPA (Ingenuity^®^ System, http://www.ingenuity.com) to combine filtering and miRNA matching to mRNA targets in order to provide insight into the potential biological effects of miRNA and to predict their role within target pathways. This software uses TarBase version 7 [42], which houses a manually curated collection of experimentally tested miRNA targets. The input dataset was compared to all available biological pathways provided by the Kyoto Encyclopaedia of Genes and Genomes (KEGG) [43], with significantly influenced pathways identified (P < 0.05) and a False Discovery Rate of 5%. The database was searched with the full names of each murine miRNA as per the ThermoFisher Scientific product information and miRBase version 21: mmu-miR-16-1-3p, mmu-miR-21a-3p, mmu-miR-146a-5p, mmu-miR-150-5p, mmu-miR-193b-3p, mmu-miR-205-5p, mmu-miR-215-5p, mmu-miR-297a-3p, mmu-miR-328-3p, mmu-miR-335-3p, mmu-miR-467a-5p, mmu-miR-486a-5p, mmu-miR-685, mmu-miR-1949, and rno-miR-10b-5p.

### RT-qPCR analysis

RT-qPCR results often depend on the quality of the endogenous control genes chosen, ideally demonstrating gene expression that is constant and highly abundant across tissues and cell types, and across changing biological conditions or treatments. To verify these miRNA of interest highlighted by the OpenArray, four control miRNA (U6, Y1, sno-RNA-135, and sno-RNA-202—the murine miRNA from the original control panel from the OpenArray) were tested for their robustness of normalization, and their expression across all biological groups and sample types. RT-qPCR analysis was performed using the Taqman gene expression master mix, following recommended protocols. The starting RNA concentration was set at 10 ng and each sample was tested in duplicate. Taqman small RNA assays for the miRNA of interest, i.e. hsa-miR-146a, hsa-miR-150, hsa-miR-205, hsa-miR-486, mmu-miR-10b, mmu-miR-193b, mmu-miR-215, mmu-miR-467a, two miRNA amongst those unique to either CM or NI MV i.e. hsa-miR-590-5p and rno-miR-450, and the four controls were used. Replicates for which the SD was > 0.5 were removed. Statistical calculations were performed using a non-parametric Kruskal–Wallis test, and a two-stage post hoc test to correct for multiple comparisons by controlling the False Discovery Rate for significant results. Results were considered significant, if P < 0.05.

### Sample size calculation

A sample size calculation was performed using GraphPad Statmate software v2.0. As this formula required a known SD, the OpenArray results were used to inform the calculation of a sample size necessary for subsequent RT-qPCR verification. A significance level (alpha) of 0.05 (two-tailed) and a 90% power were chosen as the parameters for this calculation in order to increase the stringency, and decrease the chance of false-positive findings for this verification analysis. It was found that a sample size of 5 in each group was sufficient to detect a significant difference between means of ≥ 5.93.

## Results

### Experimental design

**Fig. 1:**
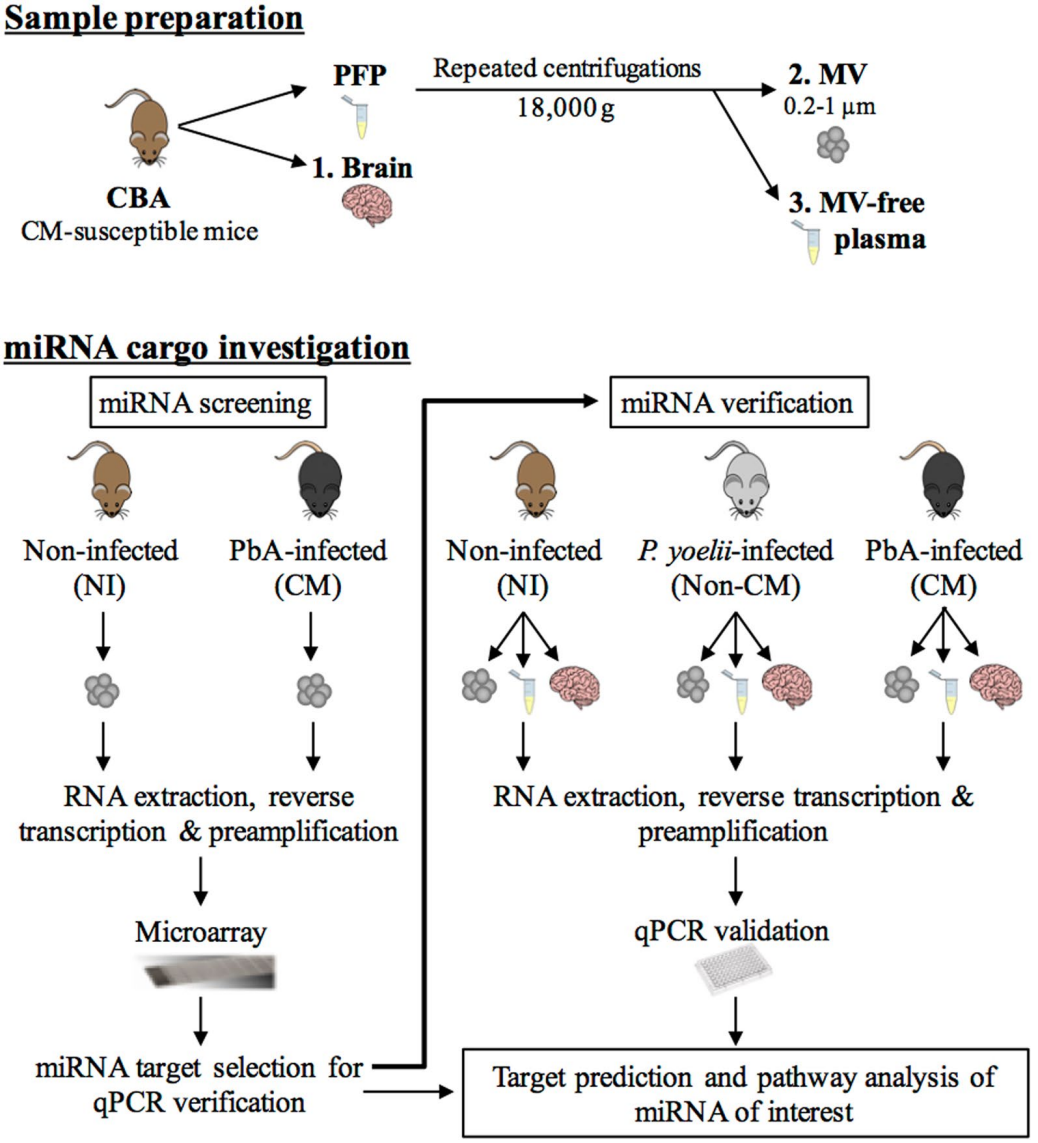
Experimental design. Graphical summary of the experimental design applied in the present study. *PFP* platelet-free plasma, *MV* microvesicle, *NI* non-infected, *CM* cerebral malaria. Images were obtained from Pixabay.com and ChemDraw (PerkinElmer)

To examine the abundance of the miRNA cargo of MV released during CM, compared with NI conditions, the OpenArray system (ThermoFisher Scientific) was used. The targets of interest found in the initial screening process were verified using qPCR analysis: examining MV, MV-free plasma, and brain tissue from NI, NCM and CM mice. miRNA of interest were evaluated in relation to CM pathogenesis using target prediction and pathway analyses. A graphical representation of the experimental design used is shown in Fig. 1.

### Distinct profile of miRNA within MV in CM compared with NI mice, using array analysis

**Fig. 2:**
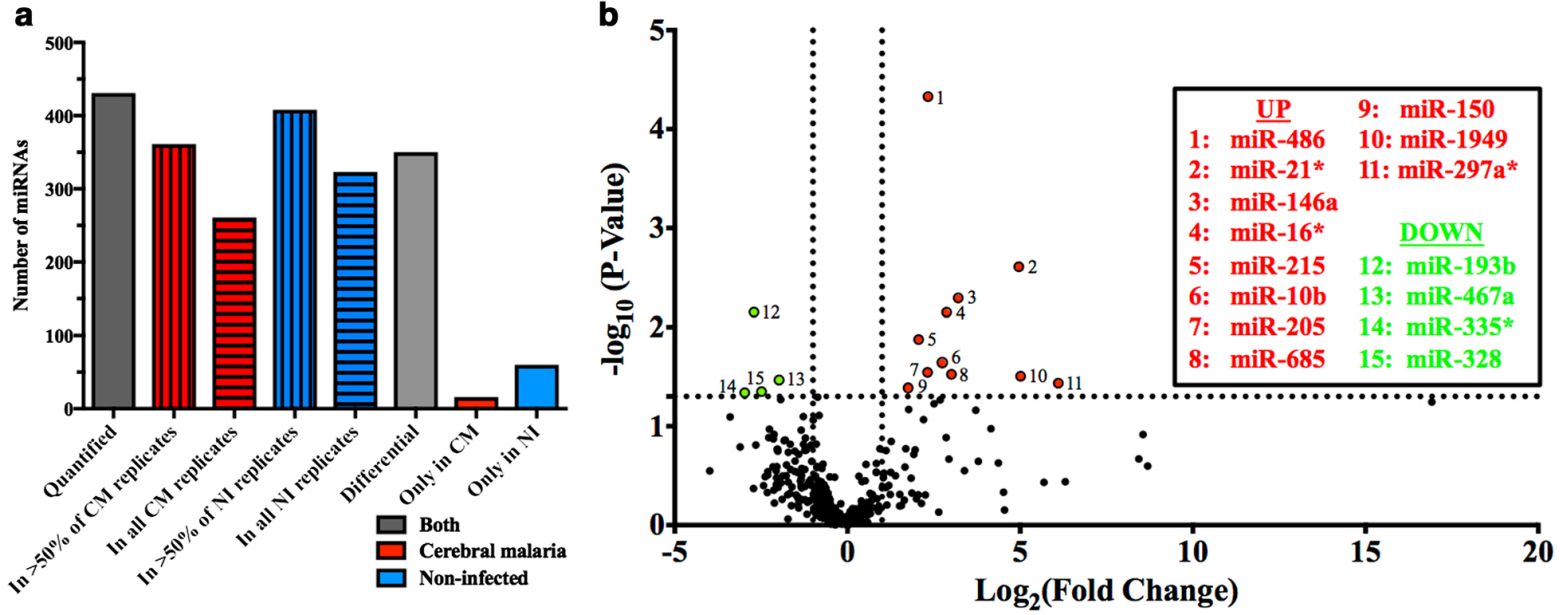
Detectable miRNA profiles in MV from NI compared with CM CBA mice. a The number of miRNA quantified in one or more biological group, and in > 50 or 100% of replicates in each biological group are shown. miRNA present in > 50% of replicates are further separated into groups of those common to both biological groups (further examined in Fig. 1b) and those expressed in one biological group only. b The volcano plot displays miRNA present in both biological groups; for each miRNA, the fold change (log_2_FC) and the significance (− log_10_P-value) are reported. P values were obtained with a student’s t test, with fewer assumptions. *Horizontal threshold line:* P = 0.05, a higher value indicates greater significance; *vertical threshold lines:* fold change = − 2 (left) and 2 (right). Highlighted in red are miRNA that are significantly increased in abundance in CM conditions, and in green, decreased

The abundance of miRNA contained in MV purified from PFP obtained from NI and CM CBA mice were compared using OpenArray^®^ analysis. For microarray analyses, three groups of NI mice and four groups of CM mice were used, with five samples pooled per group. A distinct abundance profile was observed in the MV from CM compared with NI mice. Within each biological group, approximately 60% of miRNA were detected in all replicates within a biological group, and 408 and 361 miRNA were detected in > 50% of NI (blue, vertical stripes) and CM replicates (red, vertical stripes), respectively, and were therefore studied further. 431 miRNA were quantified in at least one biological group (dark grey) and 348 miRNA were common to both biological groups (differential, light grey), while 60 and 23 miRNA were detected only in NI (blue) or CM MV (red), respectively (Fig. 2a).

### Differentially abundant miRNA play potential roles in malaria infection and inflammation

miRNA that were present in > 50% of replicates within both NI and CM groups in the microarray analysis were further analyzed to identify significant miRNA of interest (differential miRNA from Fig. 2a, n = 348). A cut-off of ± twofold was used to compare the relative expression of these miRNA between the groups, measured as a ratio of CM/NI. Figure 2b displays the results in a volcano plot, where miRNA common to both biological groups were plotted according to their fold change, and the significance of that association. Four miRNA, shown in green, showed significantly decreased abundance in CM conditions, and 11 miRNA (red) were significantly increased compared with NI conditions. These miRNA of interest were further investigated and shown to have diverse roles defined in the literature, and using DIANA miRPath software. 19 pathways were significantly regulated by the chosen miRNA of interest, as detailed in Additional file 1: Table S1 and Additional file 2: Table S2, and include those relating to Prion diseases (P = 2.2e^−20^, 7 genes controlled by 5 miRNA in pathway), regulation of actin cytoskeleton (P = 0.02, 41 genes by 8 miRNA), gap junctions (P = 0.04, 14 genes by 5 miRNA), Chagas disease (P = 0.04, 21 genes by 6 miRNA), and toxoplasmosis (P = 0.04, 22 genes by 8 miRNA). Interestingly, these miRNA strongly regulate other neurological conditions, pathways related to MV formation, or other parasitic diseases, further highlighting their potential importance in CM. Of the 15 miRNA identified by OpenArray analysis as significantly differentially abundant, eight of them were chosen for verification by RT-qPCR, according to their postulated roles in CM and other similar neurological or inflammatory conditions, as well as their regulation of genes within the malaria pathway.

### Choice of reference miRNA candidates for RT-qPCR normalization

**Fig. 3:**
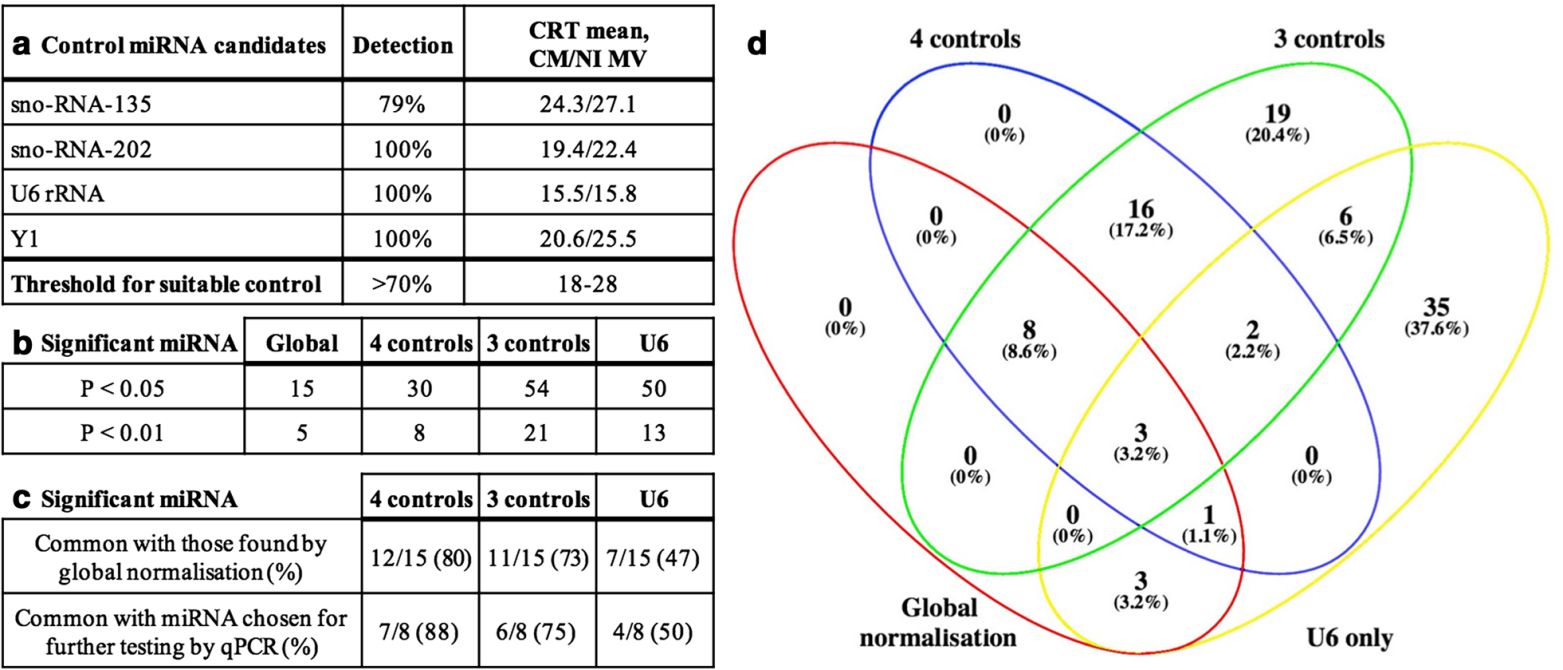
Assessing normalization strategies for miRNA relative quantification. Four candidate controls were used to normalize OpenArray results in two different combinations—one including U6 and one not, as well as U6 alone; and compared to the global normalization used for OpenArray analysis. a Each candidate control was assessed individually in terms of percentage detection and mean C_RT_ of candidate controls across all samples and biological groups tested using the OpenArray system, These candidate controls were also assessed across the four normalization strategies in terms of b number of significant miRNA, and further to this c significant miRNA from global normalization that were also significant with other normalization strategies, and, of those significant and common miRNA, how many from the list of those chosen for further validation by RT-qPCR. d Venn Diagram shows the overlap between statistically significant miRNA as determined by all normalization methods

Before validating these miRNA of interest by RT-qPCR, potential control miRNA were assessed, in order to determine the best normalization strategy to be applied to RT-qPCR results. Four candidate controls—U6, Y1, sno-RNA-135, and sno-RNA-202—were investigated on the OpenArray results. In particular, the global normalization strategy was compared to U6, and to two different combinations—one including U6 and one not (Fig. 3), as the validity of U6 as a control has previously been debated [44]. Candidate control miRNA were deemed suitable if they were detected in over 70% of samples, and if the C_RT_ values fell within the range of 18–28, as per previous rigorous testing [45, 46], and all four fulfilled these requirements (Fig. 3a). The number and type of miRNA found to be significant with the four different normalization strategies were then compared (Fig. 3b–d), and increasing numbers of significant miRNA were observed using fewer candidate control miRNA (Fig. 3b). Of the 15 miRNA found to be significantly differentially abundant using global normalization, the numbers of miRNA remaining significant, decreased with the numbers of control miRNA used in the panel, i.e., 12 miRNA with 4 controls, 11 with 3 controls and 7 with U6 alone (Fig. 3c, d). Importantly, of the eight miRNA from this list of 15 that were chosen for further validation by RT-qPCR, 7, 6 and 4 were also found to be significant when normalizing with the respectively listed groups above (Fig. 3c). Therefore, the panel of four control miRNA was chosen to normalize RT-qPCR verification results, as this strategy was found to be the most similar to global normalization.

### Significantly changed abundance of miR-146a and miR-193b in MV following *P. berghei* infection

**Fig. 4:**
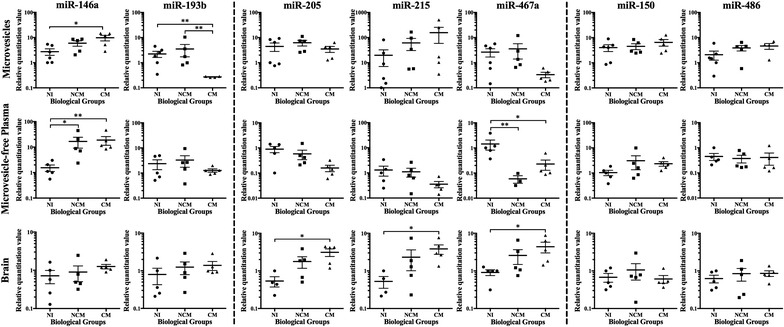
Changes in abundance of miRNA of interest in MV, MV-free plasma and brain tissue. Dot plots show the expression levels of miR-146a, miR-193b, miR-205, miR-215, miR-467a, miR-150, and miR-486 measured for MV, MV-free plasma, and brains from NI, NCM, and CM conditions, expressed as normalized values as compared to the expression of a panel of control miRNA in each case. The results are presented as follows: significant changes in MV – miR-146a and miR-193b, significant changes in the brain—miR-205, miR-215, and miR-467a, no significant changes—miR-150 and miR-486. miR-146a and miR-467a also showed significant changes in the MV-free plasma. The horizontal line denotes the mean value, and the error bars denote SEM. A nonparametric Kruskal–Wallis test was carried out. If the Kruskal–Wallis test was significant, post hoc tests—Dunn’s multiple comparisons test—were carried out. The results of these are denoted as * = 0.05–0.01, ** = 0.01–0.0001, *** ≤ 0.0001

Verification by RT-qPCR was performed on newly generated samples. Seven of the 15 miRNA selected from the OpenArray were removed from the verification due to a lack of validated targets and this will be further developed in the discussion (Table 1, miR-16*, miR-21*, miR-297a*, miR-335*, miR -328, miR-685, and miR-1949. Two additional miRNA—miR-590-5p and miR-450—were selected among those unique to either CM or NI MV for verification; however, as amplification was not adequate across all samples and biological groups, data for these two miRNA as well as for miR-10b, were not shown (Table 1). All the remaining miRNA (Table 1, miR-146a, miR-150, miR-193b, miR-205, miR-215, mir-467a, and miR-486) were tested on MV samples as per the OpenArray analysis and also on MV-free plasma and brain tissue from NI, NCM, and CM mice. This provided a more comprehensive analysis of the changes occurring during *Plasmodium* infection in order to identify those specific to neurological complications, in comparison to non-cerebral infections or non-infected status. For RT-qPCR analysis, five mice from each of the three biological groups were used, with each mouse representing an individual sample. All selected miRNA were expressed at detectable levels in all samples under normal physiological conditions, but their expression levels after the inoculation of *P. berghei* (CM) or *P. yoelii* (NCM) demonstrated altered abundance in 95% of cases. Significant changes were found in the abundance of miR-146a, miR-193b, miR-205, miR-215, and miR-467a, as shown in Fig. 4. miR-146a was significantly more abundant in MV and MV-free plasma from mice at the time of CM (P = 0.027 and 0.005, respectively), compared with that of NI mice, and in MV-free plasma from NCM mice compared with NI mice (P = 0.012), while no significant differences were observed in the brain samples. In MV samples only, miR-193b was less abundant at the time of CM, compared with both NI (P = 0.007) and NCM conditions (P = 0.007) (Fig. 4). The significant changes in miRNA abundance in MV reflected the findings of the OpenArray. Specifically, miR-146a and miR-193b were both significantly dysregulated in the MV from CM mice compared with NI-increasing 3.2- or 7.2-fold in the case of miR-146a, or decreasing 2.7- or 7.5-fold (miR-193b) in array and RT-qPCR analysis, respectively (Table 1). miR-467a was significantly less abundant in the MV-free plasma from CM mice (P = 0.050) and NCM mice (P = 0.020) compared with NI (Fig. 4). Interestingly, different profiles were observed in the brain samples: miR-205, miR-215, and miR-467a were significantly increased abundance in the brains of CM mice compared with NI (P = 0.011, 0.016, and 0.015 respectively), but no change was observed in the brains of NCM mice (Fig. 4).

**Table 1:** Choice of targets for validation by RT-qPCR from OpenArray analysis

miRNA	Significance of fold change in CM vs. NI MV	
	OpenArray	RT-qPCR
hsa-miR-328	− 2.5* ± 0.93	Not tested^a^
hsa-miR-335*	− 3.0* ± 1.13	Not tested^a^
mmu-miR-16*	2.8** ± 0.65	Not tested^a^
mmu-miR-21*	5.0** ± 0.88	Not tested^a^
mmu-miR-297a*	5.8* ± 1.60	Not tested^a^
mmu-miR-685	3.0* ± 1.00	Not tested^a^
mmu-miR-1949	5.0* ± 1.69	Not tested^a^
hsa-miR-590-5p	Unique to NI	Not validated^b^
rno-miR-450	Unique to CM	Not validated^b^
mmu-miR-10b	2.7* ± 0.85	Not validated^b^
hsa-miR-146a	3.2** ± 0.68	7.2* ± 2.74
hsa-miR-150	1.8* ± 0.64	2.7 (ns) ± 2.26
hsa-miR-205	2.3* ± 0.75	− 0.5 (ns) ± 1.89
hsa-miR-486	2.3*** ± 0.18	4.7 (ns) ± 1.45
mmu-miR-193b	− 2.7** ± 0.62	− 7.5* ± 0 62
mmu-miR-215	2.1* ± 0.55	4.6 (ns) ± 99.39^c^
mmu-miR-467a	− 2.0* ± 0.69	− 5.6 (ns) ± 0.96

The list of significantly differentially expressed miRNA in CM vs NI MV from the OpenArray analysis was compared with the results obtained by RT-qPCR analysis. Results presented as relative quantification ± SEM

P: * = 0.05–0.01, ** = 0.01–0.0001, *** ≤ 0.0001

^a^miRNA not validated by RT-qPCR due to lack of relevance to this study

^b^miRNA not validated despite being tested by RT-qPCR due to technical issues

^c^Large SEM variation due to outliers in NI and CM MV groups

### No significant change in the abundance of miR-150, miR-205, miR-215, miR-467a, and miR-486 in MV following *Plasmodium* infection

Of the seven miRNA of interest tested by RT-qPCR, miR-146a and miR-193b showed the same significant change in abundance as in the OpenArray (from Fig. 2b). A further four miRNA—miR-150, miR-215, miR-467a, and miR-486 showed the same directional change in abundance as in the OpenArray analysis, without reaching significance (Fig. 4). Comparative results for the miRNA of interest for the OpenArray and RT-qPCR analyses are displayed in Table 1. miR-205 showed a small decrease in abundance in the RT-qPCR validation experiments, compared to the significantly increased abundance in the OpenArray analysis. miR-10b, results were not shown, due to poor RT-qPCR amplification across all sample types. The direction of regulation in CM conditions was the opposite for MV and brain tissue in the case of miR-150, miR-205, miR-193b, and miR-467a. For these 4 miRNA as well as miR-146a, the direction of regulation in CM conditions was the same for MV and MV-free plasma. In all cases within the brain samples—except miR-150—miRNA abundance increased (significantly or not, in the case of specific miRNA) in CM compared with NI and NCM conditions, with NCM being an intermediate abundance level between the other two biological groups. This intermediate abundance in NCM conditions was applicable in almost all miRNA tested in MV and MV-free plasma samples as well.

### Significantly differentially abundant miRNA regulate genes with identified roles during malaria infection

**Fig. 5:**
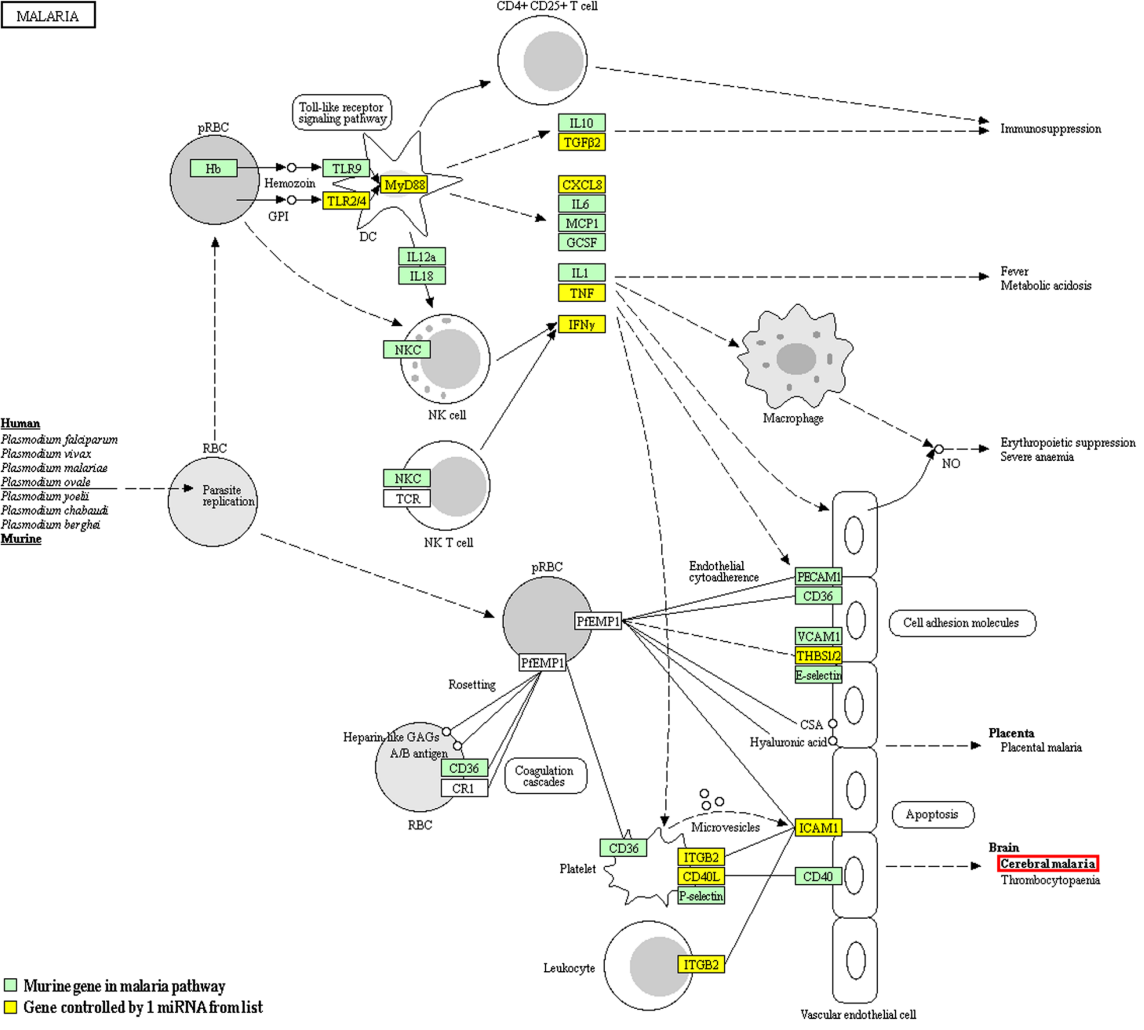
Malaria KEGG pathway. DIANA mirPath v.3 and Tarbase v.7 were used to compile lists of genes controlled by significantly differentially expressed miRNA tested using OpenArray analysis and verified by RT-qPCR analysis. Following this, the KEGG pathway for genes known to be involved in malaria infection was modified to show the role played by these miRNA. Permission has been granted for reproduction of this image. NB: some genes, including LFA1, IL8, TGFβ, and TSP are listed in the original KEGG pathway, but have been changed here to alternative but equivalent names—ITGB2, CXCL8, TGFβ2, and THBS respectively, as these gene names reflect those in the lists for the specific miRNA analyzed

Of the differentially abundant miRNA from the OpenArray analysis that were further analyzed by RT-qPCR, five significantly abundant miRNA regulate genes within the KEGG malaria pathway (Fig. 5). This figure summarizes the changes occurring during malaria infection at the gene level, and the impact this has on various cell types and molecules produced. This figure has been revised from the original KEGG pathway, with genes involved highlighted based on the miRNA of interest and their roles in regulating these genes. Within the malaria pathway, a collection of miRNA specifically regulate the groups of genes surrounding MV release, regulation of inflammatory cytokines, and cytoadherence of cells to the vascular endothelium. To summarize, three miRNA: miR-146a, miR-193b, and miR-467a, control 10 genes within the malaria pathway: CD40 ligand (CD40L, CD154), CXCL8 (IL-8), IFNγ, Integrin β2 (ITGβ2), Transforming Growth Factor β2 (TGFβ2), Thrombospondin genes (THBS1/TSP1, THBS2/TSP2), Toll-Like Receptors (TLR2, TLR4), and TNF. Further to this, many of these miRNA also have roles defined in the literature, relating to similar neuro-inflammatory conditions (more detail in Additional file 1: Table S1).

## Discussion

As miRNA research has expanded into numerous disease areas, it has become clear that expression levels of certain miRNA are altered in many diseases, including malaria [20, 24, 26]. However, despite these studies examining miRNA during malaria infection, the mechanism of action of miRNA and whether they play a pathogenic or immuno-protective role is still unclear. This study describes for the first time, the changes in miRNA content of MV during *Plasmodium* infection in mice and shows a clear dysregulation of miRNA abundance within circulating MV during experimental CM as compared with non-infected and non-CM mice.

In mice, *P. berghei* infection triggers an intricate series of events that leads to CM, including an increased release of extracellular vesicles, released from EC, platelets, and RBCs [12, 13]. miRNA have also been implicated in this process by actively regulating the immune or inflammatory response. In fact, many studies have shown that miRNA constitute a key mechanism by which MV mediate cellular communication. Specifically, during malaria infections, miRNA from pRBC MV have been shown to modulate EC permeability, affecting vascular function [27]. Furthermore, evidence of changed miRNA levels in the brains of mice with CM compared with NCM or NI mice has been reported [24, 26]. Using OpenArray technology as a screening approach, 754 miRNA targets were assessed and 15 miRNA with significantly dysregulated abundance in MV from CM mice in comparison to NI mice were detected. To verify the screened results, and to better understand miRNA changes during the severe infection, eight of these targets were selected and their abundance measured by RT-qPCR in MV, MV-free plasma, and brain tissue from CM and NI mice. The remaining seven miRNA were not tested as they had no validated murine targets following IPA analysis and/or few or no publications were available examining them. In contrast, the remaining miRNA of interest chosen for RT-qPCR verification did have validated murine targets, as well as relevant roles related to neuropathologies and inflammatory conditions similar to CM (Additional file 2: Table S2). To assess the results regarding their specificity to the malarial neurological syndrome, samples obtained from NCM mice were also analyzed.

Upon verification, two miRNA were confirmed as significantly dysregulated in CM MV: miR-146a and miR-193b, suggesting a role for these miRNA in cerebral pathology, as the same significant changes were not observed in NCM mice [47]. miR-146a was significantly over-abundant in CM MV and MV-free plasma compared to NI, but not in brain tissue—although the same trend in abundance shift was observed. An increased abundance of miR-146a is triggered by inflammatory factors including IL-1 and TNF [48], and dysregulates several targets, the majority of which are involved in toll-like receptor pathways, triggering a cytokine response, or in the innate immune system [48, 49], and these changes are also observed in CM [50]. Within the malaria pathway, miR-146a suppresses the CD40L, CXCL8, IFNγ, TLR2, TLR4, and ITGβ2 genes. Together with miR-155 and miR-21, miR-146a operates in a negative feedback loop to specifically calibrate inflammatory responses [51]. The role this miRNA plays within inflammatory conditions [49] has been researched extensively, identifying miR-146a as a biomarker for sepsis [52] and prion infections [53]. Notably, in the latter, miR-146a was part of a miRNA signature within exosomes from prion-infected neuronal cells. Finally, miR-146a has also been implicated in the overactive response to infection, therefore has previously been proposed to have potential as a therapeutic target [54].

Remarkably, miR-193b was significantly decreased in CM MV as compared not only to NI, but also NCM MV. Critically, no similar significant difference was observed in the MV-free plasma or brain tissue, though the opposite trend in abundance shift was observed in the latter. miR-193b plays a role in the TGFβ2 signaling pathway [55], and in monocyte-macrophage differentiation [56], both of which have been explored in relation to CM [50]. Within the malaria pathway, miR-193b promotes the THBS1, THBS2, and TGFβ2 genes; and regulates apoptosis by targeting myeloid leukaemia cell differentiation protein 1, among others [57]. Notably, miR-193b is a predictor of mortality in several other severe inflammatory conditions with associated neurotropic complications. Specifically, miR-193b is decreased in abundance in amyotrophic lateral sclerosis [58], and in sepsis [59], for which it has been identified as a biomarker.

Interestingly, miR-146a and miR-467a were significantly increased and decreased, respectively, in both CM and NCM MV-free plasma compared to NI. Therefore, these miRNA are likely to be indicative of a severe malaria infection, rather than specific to neurological complications. In contrast to the changes observed in MV, miR-205, miR-215, and mir-467a were increased only in the brains of CM compared with NI mice, or NCM mice. These data support the observation that particular miRNA may be detectable only in specific samples, suggesting a preferential expression from specific cell types [60, 61].

The differences in abundance profiles observed in the three different sample types analyzed provide further evidence that miRNA appear to be selectively expressed in the circulation, whether in the MV-free plasma itself, or expressed on the surface of or packaged within circulating MV. Specifically, the directional change in abundance of these miRNA of interest in MV-free plasma in CM conditions more closely reflected the changes within MV, compared with the opposite changes within brain tissue. In examining further the brain, all but one miRNA (miR-150) showed increased abundance in the brains of CM mice, as compared with their NI counterparts; interestingly their NCM counterparts demonstrated intermediate levels of expression, further demonstrating the difference in the degree of in severity between the two infections.

In line with previous studies comparing plasma or serum, and MV, MV-free plasma samples showed a more variable expression pattern for the miRNA of interest [62]. With the MV portion of the MV-free plasma removed, remaining miRNA are likely to be stable circulating extracellular miRNA secreted from cells, or present in other types of vesicles not tested in the current analysis—such as exosomes, as both of these sources have previously been identified in other diseases or conditions [27, 63]. Previous studies have identified miRNA present in a range of fluids and biological specimens or tissue types. Among these profiles, some highly abundant miRNA were common to multiple sample types, while others were enriched only in particular conditions [61], and the same phenomenon was observed in this study. The difference in abundance of particular miRNA in particular sample types here indicates the variety of the roles played by certain miRNA in specific locations or conditions.

Those miRNA validated by RT-qPCR and displaying significant differences in abundance during CM compared with NI conditions were examined within the KEGG pathway for malaria (Fig. 5), which highlights evidence of miRNA regulation relating to many of the critical components in CM development. Specifically, during severe *Plasmodium* infection, miR-146a and/or miR-193b regulate functions such as production of inflammatory cytokines (also involving miR-467a), recruitment of macrophages, increased nitric oxide production, disruption of neuronal function, as well as modulation of the expression of surface molecules controlling interactions between circulating cells and endothelial cells [4, 64].

Of importance to this work are the recent improvements in the understanding of the role of miRNA in the regulation of molecular and cellular networks associated with inflammation [65]. Consequentially, miRNA potential as biomarkers of inflammatory processes and infectious diseases has become increasingly evident [65]. In this context, further investigations of miRNA contained in MV (such as miR-146a and miR-193b) or circulating in the MV-free plasma (such as miR-146a and miR-467a), as well as the remaining miRNA of interest from the OpenArray not yet verified by RT-qPCR, such as miR-328 and miR-335*, are necessary to assess whether these miRNA are biomarkers of severity or mortality. Detailed examination of the kinetics of these miRNA in MV over the course of infection could reveal novel candidate markers for the early prognosis or diagnosis of CM. Furthermore, the evaluation of their abundance in human paediatric samples as well as functional studies using in vitro CM models to establish their cellular source and targets, will help to understand the clinical potential of these miRNA and their role in CM pathogenesis. It is also important to note that the OpenArray identified a significant number of miRNA expressed only in CM or NI MV, that warrant further investigation as to their biological relevance. In investigating for the first time the miRNA content of murine MV during CM, these results indicate that the content of plasma MV is affected during the severe infection and should be considered in the understanding of CM pathogenesis.

## Additional files

**Additional file 1: Table S1.** Pathways significantly regulated by miRNA of interest. The list of significantly differentially expressed miRNA from the OpenArray analysis was analyzed using miRPath software to determine the significantly regulated pathways they controlled. The KEGG pathway identification and P value of each pathway is shown, as well as the number of genes within each pathway controlled by a number of miRNA from our list of miRNA of interest.

**Additional file 2: Table S2.** Significantly differentially expressed miRNA of interest. The list of significantly differentially expressed miRNA from the OpenArray analysis was analyzed using miRPath software to determine their proposed roles and downstream targets. The directional change in regulation of each miRNA in CM as compared to NI MV is shown. These ten miRNA were chosen for further study based on their identified roles in the literature: eight are significantly differentially expressed, and two were selected from the populations of miRNA unique to each biological group. Here, P value thresholds of significance as defined previously.
