# Supplementary material for: Differential plasma microvesicle and brain profiles of microRNA in experimental cerebral malaria
Source: Malar J. 2018 May 11;17:192. doi: 10.1186/s12936-018-2330-5 (PMC5946432; doi:10.1186/s12936-018-2330-5)
Supplement: Supplementary file 1 — Additional file 1: Table S1. Pathways significantly regulated by miRNA of interest. The list of significantly differentially expressed miRNA from the OpenArray analysis was analyzed using miRPath software to determine the significantly regulated pathways they controlled. The KEGG pathway identification and P value of each pathway is shown, as well as the number of genes within each pathway controlled by a number of miRNA from our list of miRNA of interest. [file 12936_2018_2330_MOESM1_ESM.docx]

**Table S1**

| **#** | **KEGG pathway** | **p-value** | **# genes** | **# miRNA** | **miRNA controlling pathway** |
| --- | --- | --- | --- | --- | --- |
| 1 | Prion diseases (mmu05020) | 2.22e^-20^ | 7 | 5 | mmu-miR-16*, 146a, 150, 193b, 328 |
| 2 | Lysine degradation (mmu00310) | 8.61e^-6^ | 16 | 7 | mmu-miR-10b, 16*, 146a, 150, 193b, 215, 467a |
| 3 | Valine, leucine and isoleucine degradation (mmu00280) | 0.015 | 14 | 5 | mmu-miR-146a, 150, 193b, 215, 328 |
| 4 | Estrogen signaling pathway (mmu04915) | 0.015 | 20 | 8 | mmu-miR-146a, 150, 193b, 205, 215, 328, 335*, 467a |
| 5 | Regulation of actin cytoskeleton (mmu04810) | 0.025 | 41 | 8 | mmu-miR-16*, 146a, 150, 193b, 205, 328, 335*, 467a |
| 6 | Neurotrophin signaling pathway (mmu04722) | 0.038 | 25 | 6 | mmu-miR-146a, 150, 193b, 205, 215, 467a |
| 7 | Renal cell carcinoma (mmu05211) | 0.042 | 15 | 4 | mmu-miR-146a, 150, 193b, 467a |
| 8 | Pyruvate metabolism (mmu00620) | 0.042 | 11 | 5 | mmu-miR-146a, 150, 193b, 215, 328 |
| 9 | Fatty acid metabolism (mmu01212) | 0.042 | 11 | 5 | mmu-miR-16*, 146a, 150, 193b, 328 |
| 10 | Dorso-ventral axis formation (mmu04320) | 0.042 | 9 | 5 | “ |
| 11 | Gap junction (mmu04540) | 0.042 | 14 | 5 | mmu-miR-146a, 150, 193b, 328, 467a |
| 12 | Terpenoid backbone synthesis (mmu00900) | 0.042 | 8 | 6 | mmu-miR-16*, 146a, 150, 193b, 215, 328 |
| 13 | GABAergic synapse (mmu04727) | 0.042 | 19 | 6 | mmu-miR-146a, 150, 193b, 215, 328, 467a |
| 14 | Chagas disease (African trypanosomiasis) (mmu05142) | 0.042 | 21 | 6 | “ |
| 15 | Central carbon metabolism in cancer (mmu05230) | 0.042 | 12 | 6 | mmu-miR-146a, 150, 193b, 205, 328, 467a |
| 16 | Bacterial invasion of epithelial cells (mmu05100) | 0.042 | 16 | 7 | mmu-miR-16*, 146a, 150, 193b, 205, 328, 467a |
| 17 | Toxoplasmosis (mmu05145) | 0.042 | 22 | 8 | mmu-miR-16*, 146a, 150, 193b, 205, 215, 328, 467a |
| 18 | Protein processing in endoplasmic reticulum (mmu04141) | 0.042 | 37 | 9 | mmu-miR-16*, 146a, 150, 193b, 205, 215, 328, 335*, 467a |
| 19 | Steroid biosynthesis (mmu00100) | 0.048 | 3 | 3 | mmu-miR-146a, 193b, 215 |
