# Supplementary material for: Differential plasma microvesicle and brain profiles of microRNA in experimental cerebral malaria
Source: Malar J. 2018 May 11;17:192. doi: 10.1186/s12936-018-2330-5 (PMC5946432; doi:10.1186/s12936-018-2330-5)
Supplement: Supplementary file 2 — Additional file 2: Table S2. Significantly differentially expressed miRNA of interest. The list of significantly differentially expressed miRNA from the OpenArray analysis was analyzed using miRPath software to determine their proposed roles and downstream targets. The directional change in regulation of each miRNA in CM as compared to NI MV is shown. These ten miRNA were chosen for further study based on their identified roles in the literature: eight are significantly differentially expressed, and two were selected from the populations of miRNA unique to each biological group. Here, P value thresholds of significance as defined previously. [file 12936_2018_2330_MOESM2_ESM.docx]

**Table S2**

| *miRNA of*  *interest* | *Change in abundance*  *in CM* | *Pathways involved, or known roles* |
| --- | --- | --- |
| *hsa-miR-146a* | Up | Regulates IL-1β in cytokine-induced tolerance [1]; serum indicator for dengue infection [2]; suppresses cellular immune response during Japanese encephalitis [3]; induces apoptosis [4]; associated with Alzheimer’s disease [5]  **Genes in malaria pathway:** suppresses CD40LG [6], CXCL8 [7, 8], IFNɣ [9], TLR2 [10], TLR4 [11], and ITGB2 [12] |
| *hsa-miR-150* | Up | In CM mouse brains [13], monocytes [14] monocyte-derived microvesicles [15], T cell exosomes [16], and platelets [17]; promotes proliferation and apoptosis [18]; immune suppression function [19]; altered during immune response and disease conditions [20] such as ulcerative colitis [21] |
| *hsa-miR-205* | Up | Regulates apoptosis [22, 23], VEGF-mediated promotion of ovarian cancer cell invasion [24], biomarker for cervical cancer [25] and influenza B [26], potential extracellular vesicle-derived biomarker for pulmonary TB [27] |
| *hsa-miR-328* | Down | Down-regulated in *H. pylori*-associated gastric cancer [28]; regulation of ATP-binding cassette transporter ABCG2 [29], increased function of macrophages and neutrophils during infection [30], inhibits proliferation [31]  **Genes in malaria pathway:** increases Thbs1 [32] |
| *hsa-miR-335** | Down | Regulates Parkinson’s disease susceptibility [33], reduced in , ulcerative colitis [34], osteoarthritis [35], Crohn’s disease [36], and during HIV infection [37]  **Genes in malaria pathway:** increases Thbs1 and Thbs2 [32], and TLR4 |
| *hsa-miR-486* | Up | Regulates normal erythropoiesis and enhances growth [38]; targets NFκβ [39] and PIK3R1 [40], characterised in serum exosomes [41, 42], including in hypoxic states, increased in Huntington’s disease [43] and MS patients [44]  **Genes in malaria pathway:** suppresses CD40 [45] and TLR2 [46] |
| *mmu-miR-10b* | Up | Related to brain pathology in Huntington’s [43] and Parkinson’s diseases [47], part of hypoxic signature of miRNA in glioblastoma [48], identified in exosomes [49]; upregulated by proinflammatory cytokines [50]  **Genes in malaria pathway:** suppresses IL12A [46] |
| *mmu-miR-16** | Up | Role in embryo implantation [51] |
| *mmu-miR-21** | Up | Important role in induction of asthmatic inflammation [52] and seizure deterioration in epilepsy [53]; upregulated during Toxoplasma infection [54] |
| *mmu-miR-193b* | Down | Inhibits TGFβ2 signalling pathway [55], induces IL10-expressing monocytes [56], characterised in PBMC’s [57], role in monocyte-macrophage differentiation [58], potential sepsis biomarker [59], down-regulated in Alzheimer’s disease [60] and ALS [61]  **Genes in malaria pathway:** increases Thbs1 and Thbs2 [62], and TGFβ2 [63] |
| *mmu-miR-215* | Up | Promotes apoptosis [64], hypoxia-induced [65], inflammatory mediator in cystic fibrosis lung epithelial cells [66], identified in exosomes [67] |
| *mmu-miR-297a** | Up | *No published results* |
| *mmu-miR-467a* | Down | Regulates apoptosis [68] and cell survival [69]  **Genes in malaria pathway:** increases TNF [70] |
| *mmu-miR-685* | Up | *No published results* |
| *mmu-miR-1949* | Up | Potential inducer of bladder cancer [71] |
| *hsa-miR-590-5p* | Only in  NI | Potential biomarker in coronary artery disease [72], modifies cell proliferation, differentiation and migration through TGFβ signalling pathway [73], biomarker for dengue [2] and Alzheimer’s disease patients [74], identified in monocytes [75], and in T cells during MS [76] |
| *rno-miR-450* | Only in CM | Inflammatory marker in diabetes [77], targets interferon regulatory factor 2 in lung cancer [78] |

1. Nahid MA, Satoh M, Chan EK: Interleukin 1β-responsive microRNA-146a is critical for the cytokine-induced tolerance and cross-tolerance to toll-like receptor ligands. *Journal of Innate Immunity* 2015, 7(4):428-440.

2. Ouyang X, Jiang X, Gu D, Zhang Y, Kong SK, Jiang C, Xie W: Dysregulated serum miRNA profile and promising biomarkers in dengue-infected patients. *International Journal of Medical Sciences* 2016, 13(3):195-205.

3. Sharma N, Verma R, Kumawat KL, Basu A, Singh SK: miR-146a suppresses cellular immune response during Japanese encephalitis virus JaOArS982 strain infection in human microglial cells. *Journal of Neuroinflammation* 2015, 12:30.

4. Xu B, Huang Y, Niu X, Tao T, Jiang L, Tong N, Chen S, Liu N, Zhu W, Chen M: Hsa-miR-146a-5p modulates androgen-independent prostate cancer cells apoptosis by targeting ROCK1. *Prostate* 2015, 75(16):1896-1903.

5. Jiang W, Zhang Y, Meng F, Lian B, Chen X, Yu X, Dai E, Wang S, Liu X, Li X *et al*: Identification of active transcription factor and miRNA regulatory pathways in Alzheimer's disease. *Bioinformatics* 2013, 29(20):2596-2602.

6. Chen T, Li Z, Jing T, Zhu W, Ge J, Zheng X, Pan X, Yan H, Zhu J: MicroRNA-146a regulates the maturation process and pro-inflammatory cytokine secretion by targeting CD40L in oxLDL-stimulated dendritic cells. *FEBS Lett* 2011, 585(3):567-573.

7. Bhaumik D, Scott GK, Schokrpur S, Patil CK, Campisi J, Benz CC: Expression of microRNA-146 suppresses NF-kappaB activity with reduction of metastatic potential in breast cancer cells. *Oncogene* 2008, 27(42):5643-5647.

8. Li Y, Vandenboom TGn, Wang Z, Kong D, Ali S, Philip PA, Sarkar FH: miR-146a suppresses invasion of pancreatic cancer cells. *Cancer Research* 2010, 70(4):1486-1495.

9. Cameron JE, Yin Q, Fewell C, Lacey M, McBride J, Wang X, Lin Z, Schaefer BC, Flemington EK: Epstein-Barr virus latent membrane protein 1 induces cellular microRNA miR-146a, a modulator of lymphocyte signaling pathways. *Journal of Virology* 2008, 82(4):1946-1958.

10. Jurkin J, Schichl YM, Koeffel R, Bauer T, Richter S, Konradi S, Gesslbauer B, Strobl H: miR-146a is differentially expressed by myeloid dendritic cell subsets and desensitizes cells to TLR2-dependent activation. *J Immunol* 2010, 184(9):4955-4965.

11. Yang K, He YS, Wang XQ, Lu L, Chen QJ, Liu J, Sun Z, Shen WF: MiR-146a inhibits oxidized low-density lipoprotein-induced lipid accumulation and inflammatory response via targeting toll-like receptor 4. *FEBS Lett* 2011, 585(6):854-860.

12. Dai R, Phillips RA, Zhang Y, Khan D, Crasta O, Ahmed SA: Suppression of LPS-induced interferon-gamma and nitric oxide in splenic lymphocytes by select estrogen regulated microRNAs: a novel mechanism of immune modulation. *Blood* 2008, 112(12):4591-4597.

13. El-Assaad F, Hempel C, Combes V, Mitchell AJ, Ball HJ, Kurtzhals JAL, Hunt NH, Mathys J-M, Grau GER: Differential microRNA expression in experimental cerebral and noncerebral malaria. *Infect Immun* 2011, 79(6):2379-2384.

14. Xiao C, Calado DP, Galler G, Thai TH, Patterson HC, Wang J, Rajewsky N, Bender TP, Rajewsky K: MiR-150 controls B cell differentiation by targeting the transcription factor c-Myb. *Cell* 2007, 131(1):146-159.

15. Zhang Y, Liu D, Chen X, Li J, Li L, Bian Z, Sun F, Lu J, Yin Y, Cai X *et al*: Secreted monocytic miR-150 enhances targeted endothelial cell migration. *Mol Cell* 2010, 39(1):133-144.

16. de Candia P, Torri A, Pagani M, Abrignani S: Serum microRNAs as biomarkers of human lymphocyte activation in health and disease. *Frontiers in Immunology* 2014, 5:43.

17. Yu S, Deng G, Qian D, Xie Z, Sun H, Huang D, Li Q: Detection of apoptosis-associated microRNA in human apheresis platelets during storage by quantitative real-time polymerase chain reaction analysis. *Journal of Blood Transfusion* 2014, 12(4):541-547.

18. Jerusalem C, Polder T, Wijers-Rouw M, Heinen U, Eling W, Osunkoya BO, Trinh P: Comparative clinical and experimental study on the pathogenesis of cerebral malaria. *Contributions to Microbiology and Immunology* 1983, 7:130-138.

19. Ghorpade DS, Holla S, Sinha AY, Alagesan SK, Balaji KN: Nitric oxide and KLF4 protein epigenetically modify class II transactivator to repress major histocompatibility complex II expression during *Mycobacterium bovis bacillus* Calmette-Guerin infection. *J Biol Chem* 2013, 288(28):20592-20606.

20. Tsitsiou E, Lindsay MA: microRNAs and the immune response. *Curr Opin Pharmacol* 2009, 9(4):514-520.

21. Fourie NH, Peace RM, Abey SK, Sherwin LB, Rahim-Williams B, Smyser PA, Wiley JW, Henderson WA: Elevated circulating miR-150 and miR-342-3p in patients with irritable bowel syndrome. *Exp Mol Pathol* 2014, 96(3):422-425.

22. Zarogoulidis P, Petanidis S, Kioseoglou E, Domvri K, Anestakis D, Zarogoulidis K: MiR-205 and miR-218 expression is associated with carboplatin chemoresistance and regulation of apoptosis via Mcl-1 and Survivin in lung cancer cells. *Cellular Signalling* 2015, 27(8):1576-1588.

23. An G, Liang S, Sheng C, Liu Y, Yao W: Upregulation of microRNA-205 suppresses vascular endothelial growth factor expression-mediated PI3K/Akt signaling transduction in human keloid fibroblasts. *Exp Biol Med* 2017, 242(3):275-285.

24. Li J, Li L, Li Z, Gong G, Chen P, Liu H, Wang J, Liu Y, Wu X: The role of miR-205 in the VEGF-mediated promotion of human ovarian cancer cell invasion. *Gynecologic Oncology* 2015, 137(1):125-133.

25. Ma Q, Wan G, Wang S, Yang W, Zhang J, Yao X: Serum microRNA-205 as a novel biomarker for cervical cancer patients. *Cancer Cell International* 2014, 14:81.

26. Peng F, He J, Loo JF, Yao J, Shi L, Liu C, Zhao C, Xie W, Shao Y, Kong SK *et al*: Identification of microRNAs in throat swab as the biomarkers for diagnosis of iInfluenza. *International Journal of Medical Sciences* 2016, 13(1):77-84.

27. Lin J, Wang Y, Zou YQ, Chen X, Huang B, Liu J, Xu YM, Li J, Zhang J, Yang WM *et al*: Differential miRNA expression in pleural effusions derived from extracellular vesicles of patients with lung cancer, pulmonary tuberculosis, or pneumonia. *Tumour Biol* 2016, 37(12):15835–15845.

28. Ishimoto T, Izumi D, Watanabe M, Yoshida N, Hidaka K, Miyake K, Sugihara H, Sawayama H, Imamura Y, Iwatsuki M *et al*: Chronic inflammation with *Helicobacter pylori* infection is implicated in CD44 overexpression through miR-328 suppression in the gastric mucosa. *J Gastroenterol* 2015, 50(7):751-757.

29. Ripperger A, Benndorf RA: The C421A (Q141K) polymorphism enhances the 3'-untranslated region (3'-UTR)-dependent regulation of ATP-binding cassette transporter ABCG2. *Biochemical Pharmacology* 2016, 104:139-147.

30. Tay HL, Kaiko GE, Plank M, Li J, Maltby S, Essilfie AT, Jarnicki A, Yang M, Mattes J, Hansbro PM *et al*: Antagonism of miR-328 increases the antimicrobial function of macrophages and neutrophils and rapid clearance of non-typeable *Haemophilus influenzae* (NTHi) from infected lung. *PLoS Pathog* 2015, 11(4):e1004549.

31. Li JR, Wang JQ, Gong Q, Fnag RH, Guo YL: MicroRNA-328 inhibits proliferation of human melanoma cells by targeting TGFβ2. *Asian Pacific Journal of Cancer Prevention* 2015, 16(4):1575-1579.

32. Schug J, McKenna LB, Walton G, Hand N, Mukherjee S, Essuman K, Shi Z, Gao Y, Markley K, Nakagawa M *et al*: Dynamic recruitment of microRNAs to their mRNA targets in the regenerating liver. *BMC Genom* 2013, 14:264.

33. Yılmaz ŞG, Geyik S, Neyal AM, Soko ND, Bozkurt H, Dandara C: Hypothesis: do miRNAs targeting the leucine-rich repeat kinase 2 gene (LRRK2) influence Parkinson's disease susceptibility? *OMICS* 2016, 20(4):224-228.

34. Ranjha R, Aggarwal S, Bopanna S, Ahuja V, Paul J: Site-specific microRNA expression may lead to different subtypes in ulcerative colitis. *PLoS One* 2015, 10(11):e0142869.

35. Tornero-Esteban P, Rodríguez-Rodríguez, L., Abásolo, L., Tomé, M., López-Romero, P., Herranz, E., González, M. A., Marco, F., Moro, E., Fernández-Gutiérrez, B., Lamas, J. R.: Signature of microRNA expression during osteogenic differentiation of bone marrow MSCs reveals a putative role of miR-335-5p in osteoarthritis. *BMC Musculoskeletal Disorders* 2015, 16:182.

36. Bai J, Li Y, Shao T, Zhao Z, Wang Y, Wu A, Chen H, Li S, Jiang C, Xu J *et al*: Integrating analysis reveals microRNA-mediated pathway crosstalk among Crohn's disease, ulcerative colitis and colorectal cancer. *Mol Biosyst* 2014, 10(9):2317-2328.

37. Devadas K, Biswas S, Haleyurgirisetty M, Ragupathy V, Wang X, Lee S, Hewlett I: Identification of host microRNAs that differentiate HIV-1 and HIV-2 infection using genome expression profiling techniques. *Viruses* 2016, 8(5):E121.

38. Wang LS, Li L, Li L, Chu S, Shiang KD, Li M, Sun HY, Xu J, Xiao FJ, Sun G *et al*: MicroRNA-486 regulates normal erythropoiesis and enhances growth and modulates drug response in CML progenitors. *Blood* 2015, 125(8):1302-1313.

39. Song L, Lin C, Gong H, Wang C, Liu L, Wu J, Tao S, Hu B, Cheng SY, Li M *et al*: miR-486 sustains NF-κB activity by disrupting multiple NF-κB-negative feedback loops. *Cell Res* 2013, 23(2):274-289.

40. Huang XP, Hou J, Shen XY, Huang CY, Zhang XH, Xie YA, Luo XL: MicroRNA-486-5p, which is downregulated in hepatocellular carcinoma, suppresses tumor growth by targeting PIK3R1. *FEBS Journal* 2015, 282(3):579-594.

41. Rekker K, Saare M, Roost AM, Kubo AL, Zarovni N, Chiesi A, Salumets A, Peters M: Comparison of serum exosome isolation methods for microRNA profiling. *Clinical Biochemistry* 2014, 47(1-2):135-138.

42. Salomon C, Guanzon D, Scholz-Romero K, Longo S, Correa P, Illanes SE, Rice GE: Placental exosomes as early biomarker of preeclampsia - potential role of exosomal microRNAs across gestation. *J Clin Endocrinol Metab* 2017.

43. Hoss AG, Lagomarsino VN, Frank S, Hadzi TC, Myers RH, Latourelle JC: Study of plasma-derived miRNAs mimic differences in Huntington's disease brain. *Movement Disorders* 2015, 300(14):1961-1964.

44. Regev K, Healy BC, Khalid F, Paul A, Chu R, Tauhid S, Tummala S, Diaz-Cruz C, Raheja R, Mazzola MA *et al*: Association between serum microRNAs and magnetic resonance imaging measures of multiple sclerosis severity. *JAMA Neurol* 2017, 74(3):275-285.

45. Mees ST, Mardin WA, Sielker S, Willscher E, Senninger N, Schleicher C, Colombo-Benkmann M, Haier J: Involvement of CD40 targeting miR-224 and miR-486 on the progression of pancreatic ductal adenocarcinomas. *Annals of Surgical Oncology* 2009, 16(8):2339-2350.

46. Zhang X, Zuo X, Yang B, Li Z, Xue Y, Zhou Y, Huang J, Zhao X, Zhou J, Yan Y *et al*: MicroRNA directly enhances mitochondrial translation during muscle differentiation. *Cell* 2014, 158(3):607-619.

47. Hoss AG, Labadorf A, Beach TG, Latourelle JC, Myers RH: microRNA profiles in Parkinson's disease prefrontal cortex. *Frontiers in Aging Neuroscience* 2016, 8:36.

48. Agrawal R, Pandey P, Jha P, Dwivedi V, Sarkar C, Kulshreshtha R: Hypoxic signature of microRNAs in glioblastoma: insights from small RNA deep sequencing. *BMC Genom* 2014, 15:686.

49. Zhou X, Jiao Z, Ji J, Li S, Huang X, Lu X, Zhao H, Peng J, Chen X, Ji Q *et al*: Characterization of mouse serum exosomal small RNA content: the origins and their roles in modulating inflammatory response. *Oncotarget* 2017, 8(26):42712-42727.

50. Chen L, Al-Mossawi MH, Ridley A, Sekine T, Hammitzsch A, de Wit J, Simone D, Shi H, Penkava F, Kurowska-Stolarska M *et al*: miR-10b-5p is a novel Th17 regulator present in Th17 cells from ankylosing spondylitis. *Ann Rheum Dis* 2017, 76(3):620-625.

51. Zhao X, Hao H, Du W, Zhu H: Effect of vitrification on the microRNA transcriptome in mouse blastocysts. *PLoS One* 2015, 10(4):e0123451.

52. Tang GN, Li CL, Yao Y, Xu ZB, Deng MX, Wang SY, Sun YQ, Shi JB, Fu QL: MicroRNAs involved in asthma after mesenchymal stem cells treatment. *Stem Cells and Development* 2016, 25(12):883-896.

53. Hu X, Fu X, Jiang AO, Yang X, Fang X, Gong G, Wei C: Multiomic analysis of mice epilepsy models suggest that miR-21a expression modulates mRNA and protein levels related to seizure deterioration. *Genetics Research* 2015, 97:e22.

54. Cong W, Zhang XX, He JJ, Li FC, Elsheikha HM, Zhu XQ: Global miRNA expression profiling of domestic cat livers following acute *Toxoplasma gondii* infection. *Oncotarget* 2017, 8(15):25599-25611.

55. Zhong Q, Wang T, Lu P, Zhang R, Zou J, Yuan S: miR-193b promotes cell proliferation by targeting Smad3 in human glioma. *Journal of Neuroscience Research* 2014, 95(5):619-626.

56. Zhang S, Guo Y, Zhang C, Gao W, Wen S, Huangfu H, Wang B: Primary laryngeal cancer-derived miR-193b induces interleukin-10-expression monocytes. *Cancer Investigation* 2015, 33(2):29-33.

57. Chang CC, Lin CC, Hsieh WL, Lai HW, Tsai CH, Cheng YW: MicroRNA expression profiling in PBMCs: a potential diagnostic biomarker of chronic hepatitis C. *Dis Markers* 2014, 2014:367157.

58. Eigsti RL, Sudan B, Wilson ME, Graff JW: Regulation of activation-associated microRNA accumulation rates during monocyte-to-macrophage differentiation. *J Biol Chem* 2014, 289(41):28433-28447.

59. Wang HJ, Zhang PJ, Chen WJ, Feng D, H. JY, Xie LX: Four serum microRNAs identified as diagnisotic biomarkers of sepsis. *Journal of Trauma and Acute Care Surgery* 2012, 73(4):850-854.

60. Zhang R, Zhang Q, Niu J, Lu K, Xie B, Cui D, Xu S: Screening of microRNAs associated with Alzheimer's disease using oxidative stress cell model and different strains of senescence accelerated mice. *Journal of Neurological Sciences* 2014, 338(1-2):57-64.

61. Li C, Chen Y, Chen X, Wei Q, Cao B, Shang H: Downregulation of microRNA-193b-3p promotes autophagy and cell survival by targeting TSC1/mTOR signaling in NSC-34 cells. *Front Mol Neurosci* 2017, 10:160.

62. Balakrishnan I, Yang X, Brown J, Ramakrishnan A, Torok-Storb B, Kabos P, Hesselberth JR, Pillai MM: Genome-wide analysis of miRNA-mRNA interactions in marrow stromal cells. *Stem Cells* 2014, 32(3):662-673.

63. Chi SW, Zang JB, Mele A, Darnell RB: Argonaute HITS-CLIP decodes microRNA-mRNA interaction maps. *Nature* 2009, 460(7254):479-486.

64. Ye M, Zhang J, Zhang J, Miao Q, Yao L, Zhang J: Curcumin promotes apoptosis by activating the p53-miR-192-5p/215-XIAP pathway in non-small cell lung cancer. *Cancer Lett* 2015, 357(1):196-205.

65. Hu J, Sun T, Wang H, Chen Z, Wang S, Yuan L, Liu T, Li HR, Wang P, Feng Y *et al*: MiR-215 is induced post-transcriptionally via HIF-Drosha complex and mediates glioma-initiating cell adaptation to hypoxia by targeting KDM1B. *Cancer Cell* 2016, 29(1):49-60.

66. Tsuchiya M, Kumar P, Bhattacharyya S, Chattoraj S, Srivastava M, Pollard HB, Biswas R: Differential regulation of inflammation by inflammatory mediators in cystic fibrosis lung epithelial cells. *Journal of Interferon and Cytokine Research* 2013, 33(3):121-129.

67. Min QH, Chen XM, Zou YQ, Zhang J, Li J, Wang Y, Li SQ, Gao QF, Sun F, Liu J *et al*: Differential expression of urinary exosomal microRNAs in IgA nephropathy. *J Clin Lab Anal* 2017.

68. Gao F, Chen S, Sun M, Mitchel RE, Li B, Chu Z, Cai J, Liu C: MiR-467a is upregulated in radiation-induced mouse thymic lymphomas and regulates apoptosis by targeting Fas and Bax. *International Journal of Biological Sciences* 2015, 11(1):109-121.

69. Zheng GX, Ravi A, Gould GM, Burge CB, Sharp PA: Genome-wide impact of a recently expanded microRNA cluster in mouse. *Proc Natl Acad Sci U S A* 2011, 108(38):15804-15809.

70. Loeb GB, Khan AA, Canner D, Hiatt JB, Shendure J, Darnell RB, Leslie CS, Rudensky AY: Transcriptome-wide miR-155 binding map reveals widespread noncanonical microRNA targeting. *Mol Cell* 2012, 48(5):760-770.

71. Wang T, Liu Y, W. Y, Zhang L, Zhang Y, Wang Z, Zhou X, Zhou H, Chu T, Hao Y *et al*: Identification of microRNAome in rat bladder reveals miR-1949 as a potential inducer of bladder cancer following spinal cord injury. *Molecular Medicine Reports* 2015, 12(2):2849-2857.

72. Ren J, Zhang J, Xu N, Han G, Geng Q, Song J, Li S, Zhao J, Chen H: Signature of circulating microRNAs as potential biomarkers in vulnerable coronary artery disease. *PLoS One* 2013, 8(12):e80738.

73. Jafarzadeh M, Soltani BM: Hsa-miR-590-5p interaction with SMAD3 transcript supports its regulatory effect on the TGFβ signaling pathway. *Cell* 2016, 18(1):7-12.

74. Sorensen SS, Nygaard AB, Christensen T: miRNA expression profiles in cerebrospinal fluid and blood of patients with Alzheimer's disease and other types of dementia - an exploratory study. *Transl Neurodegener* 2016, 5:6.

75. Long X, Li Y, Qiu S, Liu J, He L, Peng Y: MiR-582-5p/miR-590-5p targeted CREB1/CREB5-NF-kappaB signaling and caused opioid-induced immunosuppression in human monocytes. *Transl Psychiatry* 2016, 6:e757.

76. Sousa IG, do Almo MM, Simi KC, Bezerra MA, Andrade RV, Maranhao AQ, Brigido MM: MicroRNA expression profiles in human CD3+ T cells following stimulation with anti-human CD3 antibodies. *BMC Res Notes* 2017, 10(1):124.

77. Chavali V, Tyagi SC, Mishra PK: Differential expression of dicer, miRNAs, and inflammatory markers in diabetic Ins2+/- Akita hearts. *Cell Biochem Biophys* 2014, 68(1):25-35.

78. Liu F, Yu X, Huang H, Chen X, Wang J, Zhang X, Lin Q: Upregulation of microRNA-450 inhibits the progression of lung cancer *in vitro* and *in vivo* by targeting interferon regulatory factor 2. *International Journal of Molelcular Medicine* 2016, 38(1):283-290.
